# Clinical Comparative Study of Different Fundus Imaging Methods in the Acute Phase of Vogt-Koyanagi-Harada Disease

**DOI:** 10.1155/2022/5812090

**Published:** 2022-10-10

**Authors:** Hongyun Wu, Liqun Hu, Wei Tang, Guishang Chen, Jinrong Liu, Shaojun Li, Zhe Xu

**Affiliations:** Ophthalmology Department, Ganzhou People's Hospital, Ganzhou 341000, Jiangxi, China

## Abstract

**Background:**

The diversification of follow-up ophthalmic imaging examination methods, and whether there are differences in clinical characteristics of VKH at the acute stage under different images. Our study aims to compare the imaging characteristics of the acute phase of Vogt-Koyanagi-Harada disease (VKH) under different fundus imaging methods to deepen clinical knowledge.

**Methods:**

A retrospective case study was performed on fundus images of 62 eyes from 31 patients with acute phase VKH and a disease duration ≤2 months who were treated at Ganzhou People's Hospital from January 2013 to December 2020. Fundus photography (FP), optical coherence tomography (OCT), and fundus fluorescein angiography (FFA) were performed on all 62 eyes. The fundus presentations were divided into an optic disc swelling (ODS) group, a serous retinal detachment (SRD) group, and a mixed type (MT) group (both ODS and SRD), and the proportions of patients in these groups and the coincidence rate of ODS, SRD, and MT identified by the three fundus imaging modes were determined.

**Results:**

The proportion of patients with ODS was highest under FP, and the proportion of patients with MT was highest under OCT. The proportions of patients with ODS and MT in the three fundus imaging modes differed significantly (*P* < 0.05), while the proportion of patients with SRD did not (*P* > 0.05). The proportion of patients with subretinal fluid with positive OCT results was significantly higher than those with positive FFA results (81.08% vs. 59.46%) (*P* < 0.05).

**Conclusion:**

Clinically significant positive signs could be obtained for acute VKH under different imaging methods. However, compared with FP and FFA, OCT tomography is more intuitive for the observation of lesions.

## 1. Background

Vogt-Koyanagi-Harada (VKH) is an autoimmune disease characterized by granulomatous uveitis of the eyes, often accompanied by meningeal irritation, auditory abnormalities, and skin and hair abnormalities. It appears to be more prevalent in people having an Asian, Hispanic, Indian, Native American, or Mediterranean origin [1, 2]. Harada reported 5 cases of bilateral VKH in 1926 and Koyanagi reported 16 cases of VKH in 1929. Nearly 100 years have passed since this disease was first described. Although the etiology of VKH remains unclear, researchers believe that certain environmental factors (such as viral infection) cause aberrant activation of immune responses towards melanocytes in pigmented tissues through Th1 and Th17 pathways. This activation is proposed to occur in individuals with certain genetic backgrounds [3], particularly those with the HLA-DRB1^*∗*^0405 allele. Although studies on VKH-specific diagnostic biomarkers in patients with VKH have been conducted [4–6], because of the lack of large-sample, multicenter, randomized controlled studies, there are some limitations in the results. The diagnosis of VKH is mainly based on complex clinical characteristics, such as intraocular granulomatous uveitis, meningeal stimulation sign, tinnitus, hearing loss, hair loss, hair whitening, and vitiligo [7, 8]. However, fundus fluorescein angiography (FFA), optical coherence tomography (OCT), indocyanine green angiography (ICGA), and fundus photography (FP) are still effective auxiliary examination methods for evaluating ocular changes (particularly in atypical cases) and predicting outcomes during the treatment and follow-up of patients with VKH. VKH progression follows a unique pattern in which inflammation gradually spreads from the posterior to the anterior segment of the eye. VKH can be divided into the prodromal stage, acute uveitic stage, convalescent stage, and chronic/recurrent stage according to the disease onset characteristics [9]. Based on these characteristics, Professor Peizeng Yang classified VKH into the prodromal stage (within 2 weeks of uveitis), the preuveitic involvement stage (2 weeks to 2 months after uveitis), and the pre-uveitic recurrent stage (2 months after uveitis) [7] to reflect changes in histopathology and clinical characteristics with disease duration. Acute VKH presents as peripapillary choroidal thickening, swelling, exudative retinal detachment, and papillary congestion and swelling. Many studies on imaging acute VKH have been conducted, including those on FFA and OCT. Okunuki et al. classified acute VKH as serous retinal detachment (RD)-type and optic disc (OD) swelling-type based on the FFA and OCT [10]. Wang et al. also classified VKH as optic disc, choroidal, and mixed types for uveitic VKH based on integrated FFA and OCT imaging presentation [11]. Although other studies have compared the multispectral imaging (MSI) of VKH with FFA, OCT, and FP [12], MSI equipment is expensive and not available in most hospitals. In addition, there are few studies on the diagnostic strengths of FP, FFA, and OCT examinations in acute VKH. In this study, different fundus imaging methods were compared for their applicability to acute VKH.

## 2. Participants and Methods

### 2.1. Participants

This is a retrospective cross-sectional descriptive case study in which the clinical data of 62 eyes from 31 acute VKH patients who were treated at Ganzhou People's Hospital from January 2013 to December 2020 were collected. Among the 62 patients, 18 (36 eyes) were males (58.1%) and 13 (26 eyes) were females (41.9%), with a male-to-female ratio of 1.4 : 1. The patients ranged in age from 18 to 62 (41.0 ± 11.6) years. The duration from the time at which the patient first complained of decreased visual acuity or blurred vision to the time of final definitive diagnosis was regarded as the confirmed disease duration, which varied between 2 and 60 days. The mean definitive diagnosis time was 13.32 ± 16.03 days. This study complied with the Declaration of Helsinki and was reviewed and approved by the Ethics Committee of Ganzhou People's Hospital. The inclusion criteria were as follows: (1) meets the diagnostic criteria of VKH in the literature [13]: (A) No history of penetrating ocular trauma or intraocular surgery preceding the initial onset of uveitis; (B) Bilateral ocular involvement (time interval between the 2 eyes should be ≤2 weeks); (C) No evidence of infectious uveitis or accompanying systemic rheumatic diseases or evidence suggestive of other ocular disease entities; (D) Early-phase VKH disease: 1. Signs of diffuse choroiditis and exudative retinal detachment, 2. Serous retinal detachment on OCT or B-scan ultrasonography, 3. Choroidal thickening on EDI-OCT, 4. Early punctate staining and late subretinal dye pooling on FFA, and 5. Hyperfluorescence of the optic disc on FFA. (2) Complete medical history data and fundus photography, FFA, and OCT imaging data. The exclusion criteria were incomplete information or did not meet the diagnostic criteria.

### 2.2. Methods

#### 2.2.1. Ophthalmic Examination Methods

Unaided visual acuity, best corrected visual acuity (BCVA), intraocular pressure, slit lamp microscopy, FP, FFA, and OCT examinations were performed for all of the included patients. The minimum resolution angle logarithm (logMAR) chart was used for the BCVA examination during admission, and the measurements were converted to the logMAR BCVA visual acuity during standard analysis. The noncontact intraocular pressure measurement method was used to measure intraocular pressure.

#### 2.2.2. Three Methods of Image Acquisition

Three types of image acquisition methods were used. First, a nonmydriatic fundus camera (KOWA VX-10*α*; Tokyo, Japan) was used for FP. Second, a Heidelberg SPECTRALIS-HRA fundus angiography system (Heidelberg Engineering GmbH, Germany) was used for FFA. The Chinese Ophthalmological Society Fundus Disease Group Standards were used for the acquisition of FP and FFA images [14]. In FP acquisition, double visual field examination was used, with the papilla and fovea as centers. FFA image acquisition included images of the posterior retinal pole, proximal regions (vascular arch to the equator), and distal regions (anterior equator). Third, a Heidelberg SPECTRALIS-OCT (Heidelberg Engineering GmbH) was used for OCT examination. Fovea and optic disc linear scanning were used for imaging, with a scanning depth of 2 mm. Images with good quality and position were used for labeling and storage.

#### 2.2.3. Definition of Categories

FP, FFA, and OCT were used for ophthalmology examinations of 74 eyes from 37 patients. The collected images were read by 3 fundus physicians who had worked for more than 5 years. If there were differences in the reading, the opinion shared by 2 of the physicians was used. Based on the three imaging modes and disease duration, 62 eyes from 31 patients with a disease duration ≤2 months were divided into the optic disc swelling (ODS) group, serous retinal detachment (SRD) group, and mixed type (MT) group (both ODS and SRD). In FP, optic disc edema was defined as an unclear optic disc boundary, hyperemia and uplift, and shallow or no physiological depression. Retinal serous detachment was defined as multifocal blistering ridges or a rugged “hilly” appearance of the posterior retina in FP. In FFA VKH, optic disc edema was defined as follows: fluorescein leakage of blood vessels on the surface of the optic disc in early angiography, strong changes in fluorescein in the optic disc in late angiography, and unclear boundary. The FFA criteria for VKH retinal detachment were as follows: spot-like fluorescence leakage during the early stage, fluorescence dye accumulation under the retina during the late stage, delineating a lake-like appearance of neuroepithelial detachment. The OCT criteria for VKH optic disc edema involved evaluation of the thickness of the retinal nerve fiber layer around the optic disc and the total thickness of the retina around the optic disc. Retinal detachment in OCT was defined as follows: the dark area of effusion was visible under the supercortex of the retinal nerve [15, 16]. The proportions of positive patients and coincidence rates of the three methods were compared. The corresponding image characteristics were analyzed and compared based on FFA and OCT examination results.

### 2.3. Statistical Analysis

R software (version 3.6.2) was used for statistical analysis, and a difference of two-tailed*P* < 0.05 was considered to be statistically significant. The chi-square test was used for intergroup comparison of categorical variables, and the results are presented as *n* (%). For continuous variables, the *t*-test was used for normally distributed variables and data are presented as mean ± standard deviation (mean ± SD). The rank-sum test was used for nonnormally distributed variables, and the results are presented as the median (*q*1, *q*3). An intergroup difference of *P* < 0.05 was considered to be significant.

## 3. Results

### 3.1. General Information of the Participants

There were 46 study participants with a disease duration <2 weeks and 16 with a disease duration of 2 weeks to 2 months. The mean ages of the two groups were 40.4 ± 10.8 and 42.6 ± 13.8 years, respectively, and the difference between the mean ages was statistically significant (*t* = 0.44, *P* > 0.05). There were no statistically significant differences in logMAR BCVA or intraocular pressure at the time of disease onset between the two groups (Table 1).

### 3.2. Comparison of Positive Signs among the Three Imaging Methods

FP, FFA, and OCT were performed for patients with a disease duration ≤2 months. The proportions of ODS-positive patients under the three imaging methods were 30.6%, 16.1%, and 8.1%, respectively; the proportions of SRD-positive patients under the three imaging methods were 35.5%, 46.8%, and 50.0%, respectively; and the proportions of MT-positive patients under the three imaging methods were 14.5%, 22.6%, and 40.3%, respectively. The proportion of ODS-positive patients was the highest when FP was used (30.6%), the proportion of ODS-positive patients was the lowest when OCT was used (8.1%), and the difference between these proportions was statistically significant (*P* < 0.001). There was no significant difference in the proportion of SRD-positive patients when the three imaging methods were used to determine SRD (*P*=0.232). The proportion of MT-positive patients was the lowest when FP was used (14.5%), the proportion of MT-positive patients was the highest when OCT was used (40.3%), and the difference between these proportions was statistically significant (*P* < 0.001) (Tables 2 and 3).

### 3.3. Correlation of Positive Signs among Three Imaging Methods

Subretinal fluid, choroidal swelling, and papilledema are common fundus presentations of VKH. Under FFA and OCT, subretinal fluid corresponds to multilake-like dye accumulation and subfoveal and/or parafoveal liquid, respectively; choroidal swelling corresponds to radial-band hypofluorescence and choroidal folds, respectively; and papilledema corresponds to optic disc hyperfluorescence and papilledema, respectively. The corresponding relationships of these presentations under FFA and OCT were compared. The results showed that the proportion of OCT-positive cases with subretinal fluid (81.08%) was significantly higher than that of FFA-positive cases with subretinal fluid (59.46%) (*P* < 0.05) (Table 4).

## 4. Discussion

The diagnosis of VKH is mainly based on clinical diagnosis [7, 8], and the most common VKH fundus presentations are exudative retinal detachment and papilledema. FFA, OCT, ICGA, and FP are effective auxiliary examination methods for evaluating ocular changes, but they have some limitations. FP is simple to operate and has higher acceptability among patients, resulting in better adherence. Moreover, FP can intuitively and objectively record changes in fundus retinal morphology, has good objectivity, repeatability, and comparability, and is widely used in fundus examinations [17–19]. However, the effects of static image planes, image quality, refractive medium, patient cooperation, operator's technique, and device factors may result in some bias in determining the patient's condition. Fluorescence changes in blood circulation during FFA can reflect the physiological and pathological status of major blood vessels and capillaries in the retina and are used for the diagnosis, differential diagnosis, photocoagulation guidance, and visual acuity prognosis of fundus diseases [20, 21]. However, FFA is an invasive procedure associated with the following risks: in addition to common adverse reactions such as nausea, vomiting, dizziness, urticaria, and yellowing of the skin and mucous membrane, there may also be a risk of severe allergy or even shock due to allergy.

OCT is a milestone optical diagnostic method in ophthalmology as it can be used to observe precise tomography structures of the fundus and anterior segment that cannot be detected using other methods. OCT does not require any contrast agent, is noninvasive, rapid, high-resolution, and safe, but it is also expensive, so examination costs are high [22]. FP, FFA, and OCT each have their pros and cons, but are all commonly used in ophthalmology examinations.

In this study, FP, FFA, and OCT were used to classify patients with a disease duration of ≤2 months into ODS, SRD, and MT groups, and the proportions of positive patients and differences between the three examination methods were compared. The results showed that the proportions of ODS-positive patients under the three imaging methods were 30.6%, 16.1%, and 8.1%, respectively; the proportions of SRD-positive patients under the three imaging methods were 35.5%, 46.8%, and 50.0%, respectively; and the proportions of MT-positive patients under the three imaging methods were 14.5%, 22.6%, and 40.3%, respectively. The proportion of ODS-positive patients was the highest when FP was used. The chi-square test indicated there were statistically significant differences in ODS and MT among the three imaging methods (*χ*^2^ = 10.87 for ODS, *χ*^2^ = 11.28 for MT, *P* < 0.05), whereas there was no significant difference in the proportion of SRD-positive patients among the three imaging methods (*χ*^2^ = 2.92, *P* > 0.05) (Tables 2 and 3). The results also indicated that the three methods can be used to classify patients into ODS, SRD, and MT groups. FP is more intuitive and can clearly detect ODS; however, it cannot detect subretinal fluid, particularly in patients with mild subretinal fluid who tend to be overlooked in clinical practice. In addition, papilledema is one of the signs that tend to lead to delayed VKH diagnosis in the absence of other clinical signs [23, 24]. OCT can intuitively detect subretinal fluid [24] and plays an important role in the diagnosis of VKH [25–27]. In our study, the proportion of patients with ODS in the FP mode is much higher than that in the other two imaging modes, and the proportion of patients with SRD is lower than that in the other two imaging modes. Therefore, OCT is more suitable for identifying MT cases in which SRD and ODS occur simultaneously.

We also compared subretinal fluid, choroidal swelling, and papilledema, which are common presentations, between FFA and OCT (Table 4). To this end, multilake-like dye accumulation under FFA was compared with subfoveal and/or parafoveal liquid under OCT, radial-band hypofluorescence under FFA was compared with choroidal folds under OCT, and optic disc hyperfluorescence under FFA was compared with papilledema under OCT. The chi-square test was performed on the three groups. The results showed that the proportion of OCT-positive cases with subretinal fluid (81.08%) was significantly higher than the proportion of FFA-positive cases with subretinal fluid (59.46%) (*P* < 0.007) (Table 4). Therefore, subretinal fluid may not be reliably detected by FFA. In other words, multilake-like dye accumulation may not be observed under FFA even if subretinal fluid is observed under OCT. This suggests that integrated judgment should be employed in clinical practice, in which medical history, signs, and multiple fundus images are used for judgment to decrease the risk of missed diagnosis and misdiagnosis. OCT has more important examination significance as a noninvasive examination method.

This study has several limitations that warrant discussion. These include the low sample size and lack of quantitative studies on choroidal thickness. We hope that future studies will provide more comprehensive data for the analysis of different stages of VKH to deepen clinical awareness and minimize missed diagnosis and misdiagnosis.

## Figures and Tables

**Table 1:** Description of populations with different disease duration.

Factor	Total number of patients	Disease duration		*χ * ^2^/*t*	*P*
		<2 weeks	2 weeks to 2 months		
*n*	74	46	16		
*Sex*				*χ * ^2^ = 0.02	0.902
Male	46 (62.2)	26 (56.5)	10 (62.5)		
Female	28 (37.8)	20 (43.5)	6 (37.5)		
Age (years)	41.9 ± 11.2	40.4 ± 10.8	42.6 ± 13.8	*t* = 0.44	0.511
LogMAR BCVA	0.72 ± 0.45	0.67 ± 0.38	0.85 ± 0.6	*χ * ^2^ = 1.23	0.267
Intraocular pressure (mmHg)	15.13 ± 4.69	15.5 ± 5.23	13.8 ± 2.19	*χ * ^2^ = 2.12	0.146
Disease duration (days)	7 (4, 30)	5 (3, 7)	30 (27, 48.8)	54.94	<0.001

BCVA; best corrected visual acuity.

**Table 2:** Proportion of ODS-, SRD-, and MT-positive patients under FP, FFA, and OCT in patients with a disease duration ≤2 months.

Mode	Factor	*n*	Percentage (%)
*FP*	ODS	19	30.6
	SRD	22	35.5
	MT	9	14.5

*FFA*	ODS	10	16.1
	SRD	29	46.8
	MT	14	22.6

*OCT*	ODS	5	8.1
	SRD	31	50
	MT	25	40.3

FP, FFA, and OCT were performed for patients with a disease duration ≤2 months. The proportions of ODS-positive patients under the three imaging methods were 30.6%, 16.1%, and 8.1%, respectively; the proportions of SRD-positive patients under the three imaging methods were 35.5%, 46.8%, and 50.0%, respectively; and the proportions of MT-positive patients under the three imaging methods were 14.5%, 22.6%, and 40.3%, respectively. FP fundus photography; FFA fundus fluorescein angiography; OCT optical coherence tomography; ODS optic disc swelling; SRD serous retinal detachment; MT mixed type. coherence tomography; ODS optic disc swelling; SRD serous retinal detachment; MT mixed type.

**Table 3:** Differences in fundus images between methods.

	ODS		SRD		MT	
	Negative	Positive	Negative	Positive	Negative	Positive
FP	43	19	40	22	53	9
FFA	52	10	33	29	48	14
OCT	57	5	31	31	37	25
*χ * ^2^	10.87		2.92		11.29	
*P*	0.004		0.232		0.004	

There are differences in the proportions of ODS-positive patients according to each of the three fundus imaging methods. The proportion of ODS-positive patients was the highest when FP was used (30.6%) and the lowest when OCT was used (8.1%), and this difference was statistically significant (*P* < 0.001). There was no significant difference in the proportion of SRD-positive patients when the three fundus imaging methods were used to determine SRD (*P*=0.232). The proportion of MT-positive patients was the lowest when FP was used (14.5%) and the highest when OCT was used (40.3%), and this difference was statistically significant (*P* < 0.001). FP fundus photography; FFA fundus fluorescein angiography; OCT optical coherence tomography; ODS optic disc swelling; SRD serous retinal detachment; MT mixed type. The chi-square test was used for comparison of variables. An intergroup difference is present when *P* < 0.05.

**Table 4:** Comparison of clinical characteristics under FFA and OCT.

	Negative (%)	Positive (%)	Statistic	*P*
			7.277	0.007
Subretinal dye accumulation (FFA)	30 (40.54)	44 (59.46)		
Foveal subretinal fluid and parafoveal retinal fluid (OCT)	14 (18.92)	60 (81.08)		
			1.042	0.307
Optic disc hyperfluorescence (FFA)	43 (58.11)	31 (41.89)		
Papilledema (OCT)	50 (67.57)	24 (32.43)		
			1.953	0.162
Radial band hypofluorescence (FFA)	54 (72.97)	20 (27.03)		
Choroidal folds (OCT)	45 (60.81)	29 (39.19)		

The proportion of patients with subretinal fluid and positive OCT results (81.08%) was significantly higher than the proportion of patients with positive FFA results (59.46%) (*P*=0.007). FFA fundus fluorescein angiography; OCT optical coherence tomography.

## Data Availability

The datasets used and analyzed in this study are available upon reasonable request from the corresponding author.

## Abbreviations

BCVA: Best corrected visual acuity
FFA: Fundus fluorescein angiography
FP: Fundus photography
ICGA: Indocyanine green angiography
LogMAR: Minimum resolution angle logarithm
MT: Mixed type
MSI: Multispectral imaging
ODS: Optic disc swelling
OCT: Optical coherence tomography
SRD: Serous retinal detachment
VKH: Vogt-Koyanagi-Harada disease.
